# Supplementary material for: Phytochemical Screening and Antibacterial Activity of Commercially Available Essential Oils Combinations with Conventional Antibiotics against Gram-Positive and Gram-Negative Bacteria
Source: Antibiotics (Basel). 2024 May 23;13(6):478. doi: 10.3390/antibiotics13060478 (PMC11200707; doi:10.3390/antibiotics13060478)
Supplement: Supplementary file 1 [file antibiotics-13-00478-s001.zip › antibiotics-2997814-supplementary/COA - Clove bud oil.pdf]

Sigma-Aldrich.

3050 Spruce Street, Saint Louis, MO 63103, USA
Website: www.sigmaaldrich.com
Email USA: techserv@sial.com
Outside USA: eurtechserv@sial.com

Certificate of Analysis

Product Name:
Clove bud oil - natural, FCC, FG

**Product Number:**	**W232300**
**Batch Number:**	**SHBM5724**
Brand:	ALDRICH
CAS Number:	8000-34-8
MDL Number:	MFCD00130815
Quality Release Date:	01 SEP 2020
Expiration Date:	AUG 2025

Test	Specification	Result
Appearance (Color)	Pale Yellow to Light Amber-Brown	Light Yellow
Appearance (Form)	Liquid	Liquid
Refractive index at 20 ° C	1.527 - 1.535	1.534
Infrared Spectrum	Conforms to Structure	Conforms
Assay<br>(Phenols by Volume)	≥ 85.0 %	88.0 %
Solubility (Turbidity)<br>1ml/2ml 70% ethanol; clear solution<br>(may be cloudy upon dilution to 10 mls)	Clear	Clear
Angular Rotation<br>100 mm tube Neat	-1.50 - 0.00 °	-0.89 °
Specific Gravity<br>@ 25 Degrees Celsius	1.038 - 1.060	1.045
Phenols<br>(FCC Misc.)	Pass	Pass
Arsenic (As)	≤ 3 ppm	< 1 ppm
Cadmium (Cd)	≤ 1 ppm	< 1 ppm
Mercury (Hg)	≤ 1 ppm	< 1 ppm
Lead (Pb)	≤ 10 ppm	< 1 ppm
Expiration Date Period<br>5 Years	------------------------	---------------------------

Michael Grady, Manager

Sigma-Aldrich warrants, that at the time of the quality release or subsequent retest date this product conformed to the information contained in this publication. The current Specification sheet may be available at Sigma-Aldrich.com. For further inquiries, please contact Technical Service. Purchaser must determine the suitability of the product for its particular use. See reverse side of invoice or packing slip for additional terms and conditions of sale.

Sigma-Aldrich

3050 Spruce Street, Saint Louis, MO 63103, USA
Website: www.sigmaaldrich.com
Email USA: techserv@sial.com
Outside USA: eurtechserv@sial.com

Certificate of Analysis

Product Number: W232300
Batch Number: SHBM5724

Quality Control
Milwaukee, WI US

Sigma-Aldrich warrants, that at the time of the quality release or subsequent retest date this product conformed to the information contained in this publication. The current Specification sheet may be available at Sigma-Aldrich.com. For further inquiries, please contact Technical Service. Purchaser must determine the suitability of the product for its particular use. See reverse side of invoice or packing slip for additional terms and conditions of sale.
