# Supplementary material for: Phytochemical Screening and Antibacterial Activity of Commercially Available Essential Oils Combinations with Conventional Antibiotics against Gram-Positive and Gram-Negative Bacteria
Source: Antibiotics (Basel). 2024 May 23;13(6):478. doi: 10.3390/antibiotics13060478 (PMC11200707; doi:10.3390/antibiotics13060478)
Supplement: Supplementary file 1 [file antibiotics-13-00478-s001.zip › antibiotics-2997814-supplementary/COA - Eucalyptus oil.pdf]

Certificate of Analysis
Secondary reference standard

HWI group

Substance:	**Eucalyptus oil**	Specification no.:	**1876**
Article no:	**0550-15-01**		
Batch:	**HWI01627-3**		
Storage:	**amber glass ampoule, storage at ambient temperature and under inert gas**		
Expiry date[1]:	**10/2022**		

Parameter	Method*	Requirement	Result
**Characters**			
Appearance	organoleptic	colourless to slightly yellow liquid	complies
**Identity (GC)[2]**	AM0952	1,8-Cineole	complies
		α-Pinene	complies
		Limonene	complies
**Assay (GC)**			
1,8-Cineole	AM0952	---	823 mg/g

* valid version at time of testing

[1] storage in unopened, original container according to specified conditions

[2] test only performed when substance is initially tested

checked and approved	12. NOV. 2020 [signature]	12. NOV. 2020 [signature]
	Katharina Werling<br>Project leader<br>Laboratory Services	Stefanie Kempf<br>Project leader<br>Laboratory Services

HWI group

Essential oil: Eucalyptus oil
Article-no.: 0550-15-01
Batch: HWI01627-3
Calibration standard: primary reference standard 1,8-Cineol

GC method (AM0952)

Column type:	Supelcowax 10; 60 m x 0.25 mm; 0.25 µm
Carrier gas:	Helium
Flow:	1.5 ml/min
Oven:	60 °C, 5 min, 5 °C/min, 200 °C, 5 min
Detector:	220 °C
Injector:	220 °C
Injectior volume:	1 µL
Split:	1:50
Run time:	38 min

Internal standard solution

Transfer approx. 800 mg trans-Anethole into a 50.0 mL volumetric flask, dissolve in solvent and fill up to the mark with solvent.

Sample solution

Prepare two sample solutions from two different weighted portions.

Dissolve approx. 185 mg Eucalyptus oil in a 10.0 mL volumetric flask, dissolve in 5.0 mL internal standard solution and fill up to the mark with solvent.

Reference solution

Prepare two reference solutions from two different weighted portions.

Transfer approx. 46.5 mg 1,8-Cineole into a 10.0 mL volumetric flask, dissolve in 5.0 mL internal standard solution and fill up to the mark with solvent.

Figure 1: Chromatogram of Eucalyptus oil, batch HWI01627-3

Eucalyptus oil **Batch_No HWI01627**

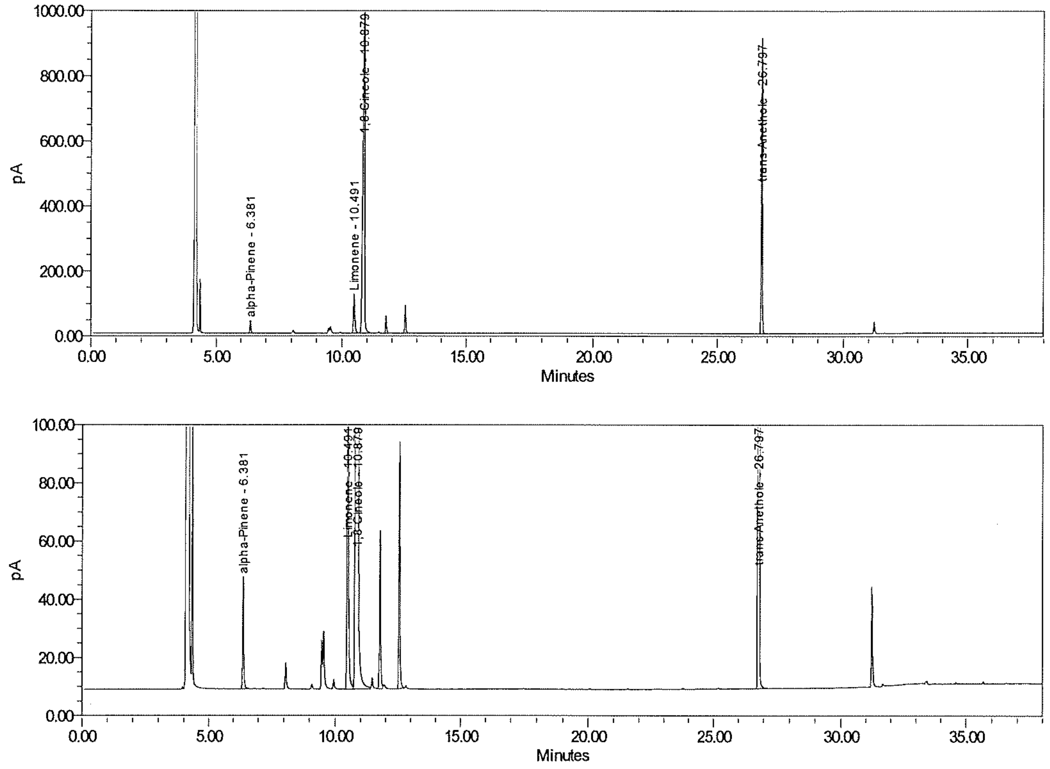

ELH20-02122-68; Eucalyptus oil; Batch_No HWI01627; Wednesday, October 28, 2020 8:45:48 PM CET; Result Id 1286

HWI group

Figure 2: Chromatogram of primary reference standard 1,8-Cineol, batch HWI00654

Reference solution 1 **Batch_No**

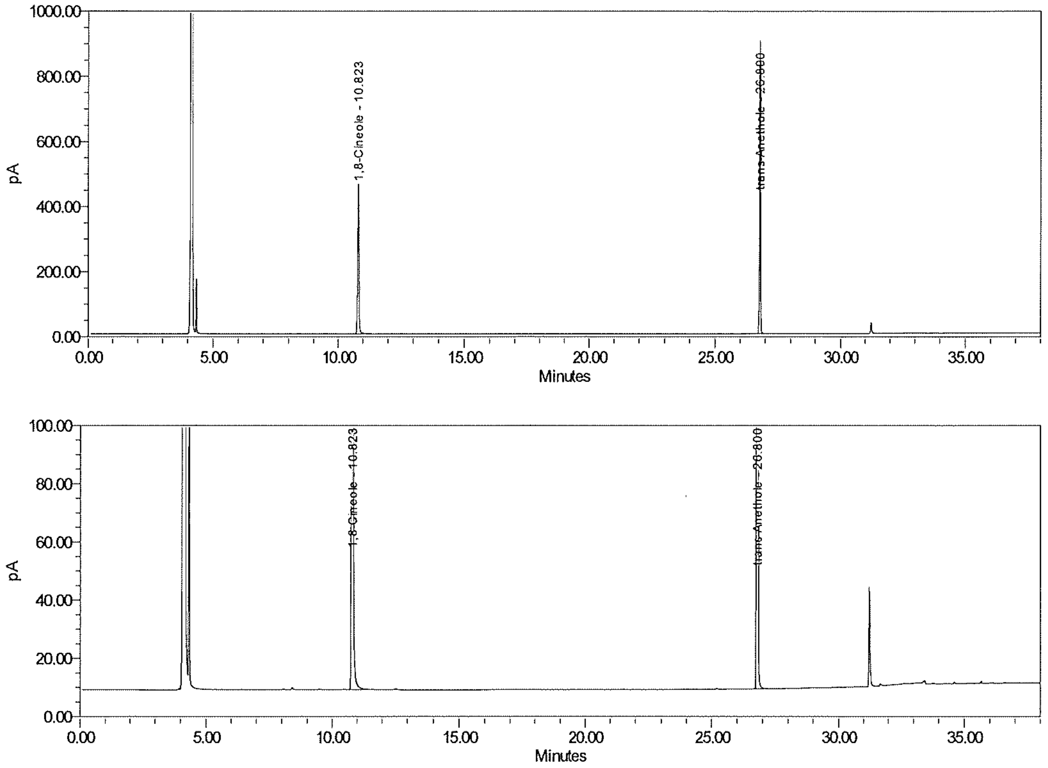

ELH20-02122-40; Reference solution 1; Batch_No ; Wednesday, October 28, 2020 2:56:16 PM CET; Result Id 1316
