# Supplementary material for: Phytochemical Screening and Antibacterial Activity of Commercially Available Essential Oils Combinations with Conventional Antibiotics against Gram-Positive and Gram-Negative Bacteria
Source: Antibiotics (Basel). 2024 May 23;13(6):478. doi: 10.3390/antibiotics13060478 (PMC11200707; doi:10.3390/antibiotics13060478)
Supplement: Supplementary file 1 [file antibiotics-13-00478-s001.zip › antibiotics-2997814-supplementary/COA - Lavender oil.pdf]

Certificate of Analysis
Secondary pharmaceutical reference standard

HWI group

Substance:	**Lavender oil**
Article no:	**0553-15-01**
Batch:	HWI01630-2
Storage:	amber glass ampoule, storage at ambient temperature and under inert gas
Expiry date[1]:	04/2022

Parameter	Method [2]	Requirement	Result
**Characters**			
Appearance	organoleptic	light yellow liquid	complies
Identity (GC) [2][3]	Ph. Eur. monograph "Lavender oil"	Linalool	complies
		Terpinen-4-ol	complies
Identity (HPTLC) [3]	external method *	Linalool	complies**
Assay (GC) [2]	Ph. Eur. monograph "Lavender oil"; calibration standard: primary reference standard Linalool batch HWI01135	---	347.69 mg/g

[1] storage in unopened, original container according to specified conditions
[2] GC conditions according to Ph. Eur. monograph
[3] test only performed when substance is initially tested
* chromatography performed by Camag, for method details see attachments
** for HPTLC chromatogram and further details see attachments

checked and approved 09. APR. 2019 [signature]

Dr. Ole Revermann

Project Leader Laboratory Services

Attachment to Certificate of Analysis – Lavender oil, HWI01630-2

Essential oil:	Lavender oil
Article-no.:	0553-15-01
Batch:	HWI01630-2
Calibration standard:	primary reference standard Linalool

GC method (Ph. Eur. Monograph "Lavender oil")

Column type:	Supelcowax 10; 60 m x 0.25 mm; 0.25 µm
Carrier gas:	Helium
Flow:	1.5 ml/min
Oven:	70 °C, 15 min, 2 °C/min, 180 °C
Detector:	220 °C
Injector:	220 °C
Injectior volume:	1µL
Split:	1:50
Run time:	70 min

Internal standard solution

Dissolve 150 mg trans-Anethole – accurately weighed - in 50 mL heptane.

Sample solution

Prepare two sample solutions from two different weighted portions.

Dissolve approx. 177 mg Lavender oil in 10 mL internal standard solution.

Reference solution

Prepare two reference solutions from two different weighted portions.

Dissolve approx. 35 mg Linalool in 10 mL internal standard solution.

Figure 1: Chromatogram of Lavender oil, batch HWI01630-2

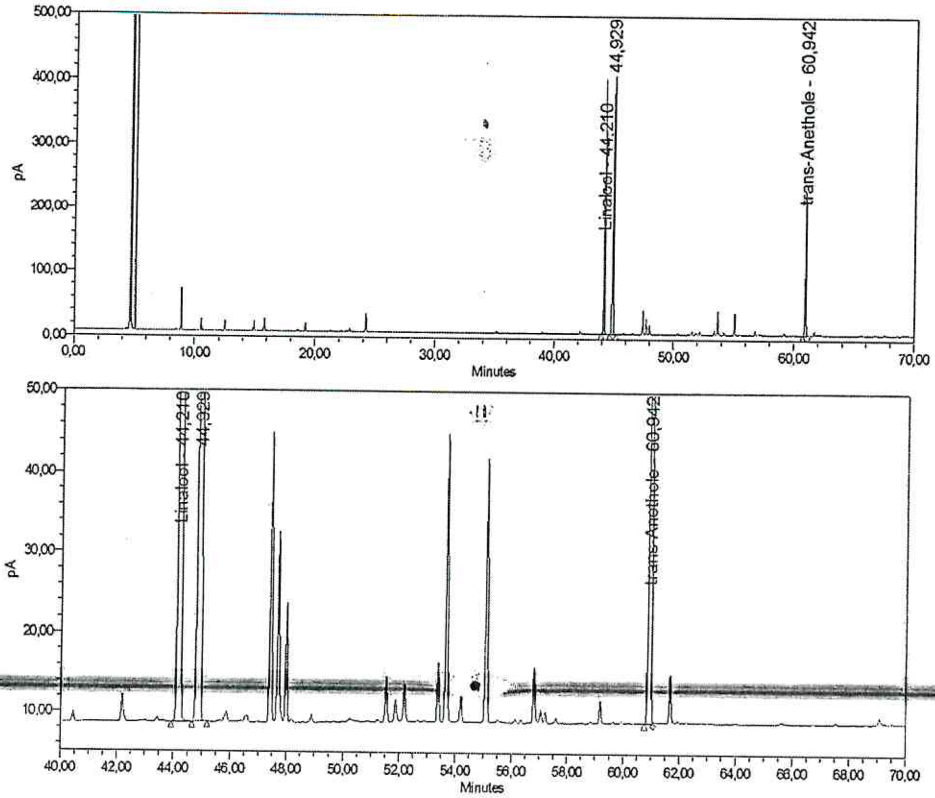

Figure 2: Chromatogram of primary reference standard Linalool, batch HWI01135-1

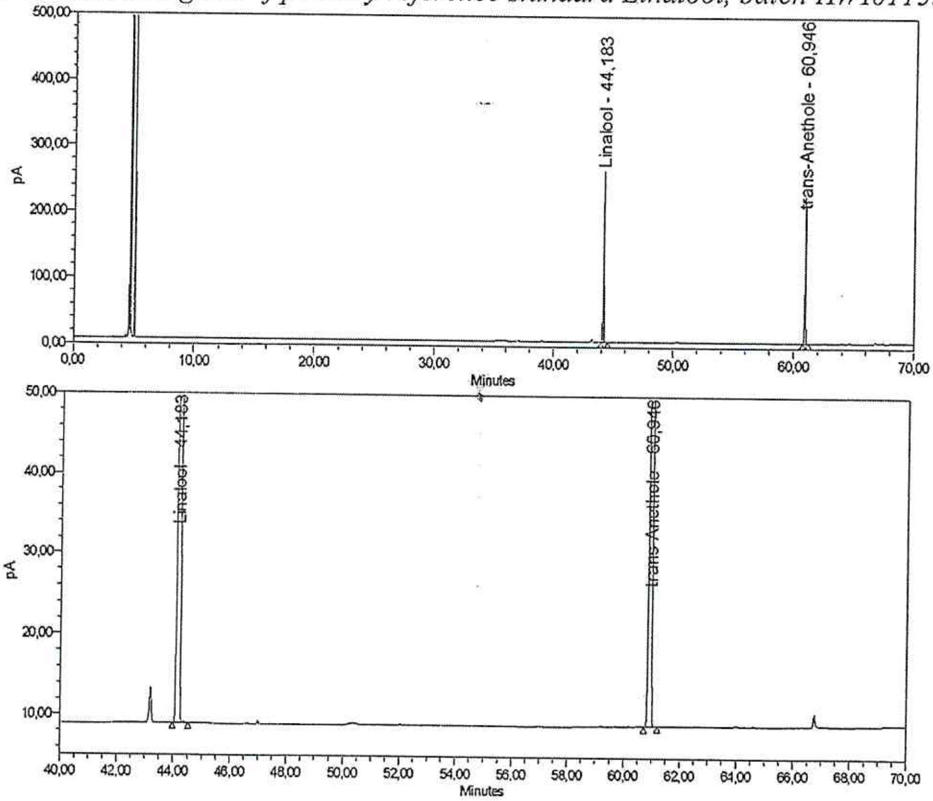

HWI group

HPTLC performed by CAMAG (Multi-method for several essential oils)

CAMAG®

HPTLC Fingerprints of Essential oil

Scope

Identification of zones seen in the HPTLC fingerprint of essential oils secondary reference standards obtained with the standard HPTLC method for identification of essential oils (submitted to Ph. Eur. for evaluation) by comparison of R_F values and colors of reference substances and matching zones in the extract.

Instrumentation

Automatic TLC Sampler (ATS 4), Automatic Developing Chamber (ADC 2), Derivatizer, TLC Plate Heater 3, TLC Visualizer 2, visionCATS 2.4 (each entry an SOP, a visionCATS instrument method, and a visionCATS Comparison Viewer file with reference images).

Samples

Essential oils are prepared as follows in toluene:

- Chamomile oil, clove oil, lemongrass oil, rose oil, and thyme oil are prepared at 5 µL/mL.
- Coriander oil, eucalyptus oil, lavender oil, neroli oil, and star anise oil are prepared at 10 µL/mL.
- Rosemary oil is prepared at 20 µL/mL.
- Turpentine oil is prepared at 100 µL/mL.

Standards

Standard solutions are prepared in toluene at the following concentrations:

- Citronellol, citronellal, and eugenol: 0.5 µL/mL
- α-bisabolol, α-bisabololoxide A, bornyl acetate, carvacrol, geranyl acetate, linalool, linalyl acetate, and terpinen-4-ol: 1 µL/mL
- Eucalyptol: 3 µL/mL
- *trans*-Anethole: 5 µL/mL
- 1.8-Cineole: 10 µL/mL
- Menthone and menthofuran: 20 µL/mL
- (R)-(+)-limonene: 120 µL/mL
- (-)-Carvone: 0.35 mg/mL
- (+)-Borneol, isoeugenyl acetate, and thymol: 0.5 mg/mL
- Menthol: 1.5 mg/mL
- Patchoulol: 2 mg/mL
- D/L-Camphor: 12.5 mg/mL
- (+)-(α)-Terpineol in methanol: 1 mg/mL

Neither α-pinene (5 µL/mL), β-pinene (5 µL/mL), nor γ-terpinene (1 µL/mL) in toluene are not detectable!

HWI group

CAMAG®

Chromatography according to standard HPTLC method for identification of essential oils (submitted to Ph. Eur. for evaluation)

Stationary phase	HPTLC Si 60 F_{254}, 20 x 10 cm (Merck).
Sample application	Bandwise application with ATS 4, 15 tracks, band length 8 mm, track distance 11.4 mm, distance from left edge 20 mm, distance from lower edge 8 mm, application volume 2 µL for test solutions and standards solutions.
Developing solvents	Ethyl acetate – toluene (5:95; *v/v*).
Development	In the ADC 2 with chamber saturation (with filter paper) for 20 min and after conditioning at 33% relative humidity for 10 min using a saturated solution of magnesium chloride $MgCl_2$.
Developing distance	70 mm (from the lower edge).
Plate drying	Drying 5 min in the ADC 2.
Documentation	With the TLC Visualizer 2 under white light and under UV 366 nm after derivatization.
Derivatization	The plate is sprayed with the Anisaldehyde reagent using the Derivatizer (nozzle: blue, spraying level: 1; spraying volume: 3 mL) and heated at 100 °C for 3 min.<br>Anisaldehyde reagent: mixture of 0.5 mL of *p*-anisaldehyde, 10 mL of glacial acetic acid, 85 mL of methanol, and 5 mL of sulfuric acid.

Lavender oil (Cat. No. 05531501 #HWI01630-1)

Documentation With the TLC Visualizer 2 under white light and UV 366 nm after derivatization.

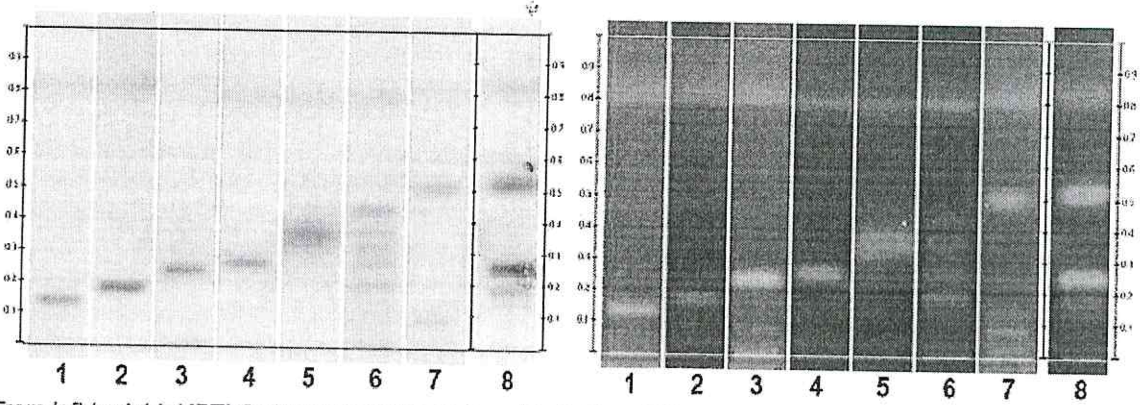

*From left to right: HPTLC chromatograms under white light and UV 366 nm after derivatization. Track 1: **(+)-α-terpineol** (R_F 0.13) **(art. N°: 83073, batch: BCBS7535V)**; 2: **(+)-borneol** (R_F 0.18) **(art. N°: 68878, batch: 14627)**; 3: **linalool** (R_F 0.24) **(art. N°: 00350190, batch: HWI01135-1)**; 4: **terpinen-4-ol** (R_F 0.26) **(art. N°: 03900590, batch: HWI00630-3)**; 5: **1.8-cineole** (R_F 0.35) **(art. N°: 00020590, batch: HWI00654-2)**; 6: **(R)-(+)-Limonene** (main zone at R_F 0.44; other zones due to degradations below) **(art. N°: 00590590, batch: HWI01323-2)**; 7: **linalyl acetate** (R_F 0.52) **(art. N°: 49599, batch: BCBS8744V)**; 8: **lavender oil (art. N°: 05531501, batch: HWI01630-1)***
