# Supplementary material for: Phytochemical Screening and Antibacterial Activity of Commercially Available Essential Oils Combinations with Conventional Antibiotics against Gram-Positive and Gram-Negative Bacteria
Source: Antibiotics (Basel). 2024 May 23;13(6):478. doi: 10.3390/antibiotics13060478 (PMC11200707; doi:10.3390/antibiotics13060478)
Supplement: Supplementary file 1 [file antibiotics-13-00478-s001.zip › antibiotics-2997814-supplementary/COA - Oregano oil.pdf]

Certificate of Analysis

Item number: 6612

Oil of origanum

natural

**batch:**	381303461		
CAS No.:	84012-24-8	print date:	16.06.2022
formula:		recommended retest date:	27.05.2024
density:	0,91	release date:	16.06.2022
molecular weight:			

	Type analysis	batch results
**Appearance**	clear, yellow to red-brown oil	complies
**Density (20 °C)**	0.915-0.975 g/cm3	0.951
**Refractive index (20 °C)**	1.500-1.520	1.5098
**Optical rotation (20 °C)**	-5° to +1°	0 °

Our products are tested for laboratory use only.
The stated information refers to the current status of product quality.
We reserve the right to implement necessary changes.

S. Lindenfelser
Quality Control

S. Ackermann
Quality Assurance

This document is computer printout and has therefore not been signed by hand.

Carl Roth GmbH + Co. KG
Schoemperlenstraße 3-5
76185 Karlsruhe

Telefon 0721/5606-0
Telefax 0721/5606-149
E-Mail: info@carlroth.de

Die Firma ist eine Kommanditgesellschaft mit Sitz in Karlsruhe, Reg. Gericht Mannheim HRA 100055. Persönlich haftende Gesellschafterin ist die Firma Roth Chemie GmbH mit Sitz in Karlsruhe, Reg. Gericht Mannheim HRB 100428. Geschäftsführer: André Houdelet
