# Supplementary material for: Phytochemical Screening and Antibacterial Activity of Commercially Available Essential Oils Combinations with Conventional Antibiotics against Gram-Positive and Gram-Negative Bacteria
Source: Antibiotics (Basel). 2024 May 23;13(6):478. doi: 10.3390/antibiotics13060478 (PMC11200707; doi:10.3390/antibiotics13060478)
Supplement: Supplementary file 1 [file antibiotics-13-00478-s001.zip › antibiotics-2997814-supplementary/COA - Peppermint oil.pdf]

3050 Spruce Street, Saint Louis, MO 63103, USA
Website: www.sigmaaldrich.com
Email USA: techserv@sial.com
Outside USA: eurtechserv@sial.com

Certificate of Analysis

Product Name:
Peppermint oil - natural, from Mentha piperita L.

**Product Number:**	**77411**
**Batch Number:**	**BCCJ7253**
Brand:	ALDRICH
CAS Number:	8006-90-4
Formula:	$C_{10}H_{16}$
Formula Weight:	136,2 g/mol
Storage Temperature:	Store at 2 - 8 °C
Quality Release Date:	16 DEC 2022

Test	Specification	Result
Appearance (Color)	Colorless to Very Faint Yellow	Colorless
Appearance (Form)	Liquid	Liquid
Purity (GC)		71.3 %
Report Result		
Refractive index at 20 ° C	1.450 - 1.470	1.460
1H NMR Spectrum	Conforms to Structure	Conforms

Dr.Reinhold Schwenninger
Quality Assurance
Buchs,Switzerland CH

Sigma-Aldrich warrants, that at the time of the quality release or subsequent retest date this product conformed to the information contained in this publication. The current Specification sheet may be available at Sigma-Aldrich.com. For further inquiries, please contact Technical Service. Purchaser must determine the suitability of the product for its particular use. See reverse side of invoice or packing slip for additional terms and conditions of sale.
