# Supplementary material for: Phytochemical Screening and Antibacterial Activity of Commercially Available Essential Oils Combinations with Conventional Antibiotics against Gram-Positive and Gram-Negative Bacteria
Source: Antibiotics (Basel). 2024 May 23;13(6):478. doi: 10.3390/antibiotics13060478 (PMC11200707; doi:10.3390/antibiotics13060478)
Supplement: Supplementary file 1 [file antibiotics-13-00478-s001.zip › antibiotics-2997814-supplementary/Supplementary Table S1 and Figures S1, S2 and S3.pdf]

antibiotics

Article

Phytochemical Screening and Antibacterial Activity of Commercially Available Essential Oils Combinations With Conventional Antibiotics Against Gram-Positive and Gram-Negative Bacteria

Supplementary Material:

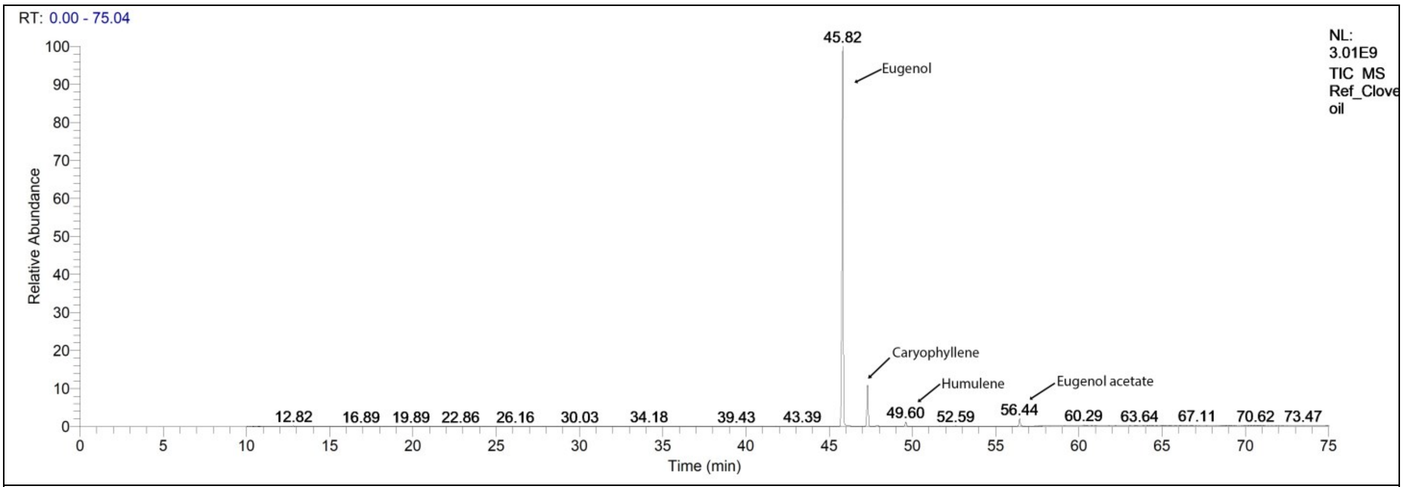

Clove Essential Oil

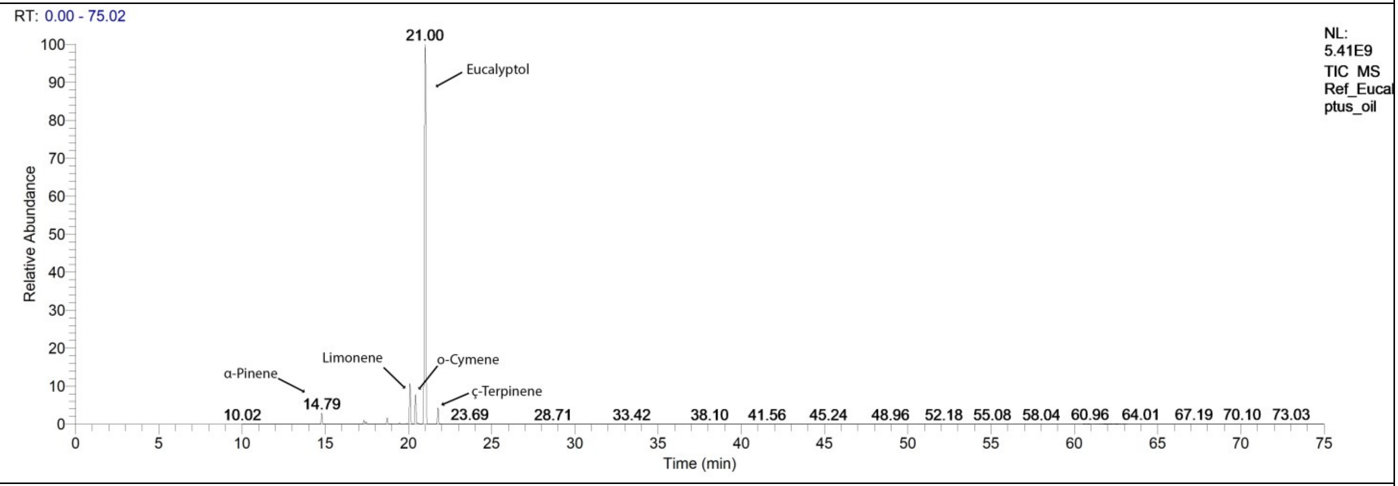

Eucalyptus Essential Oil

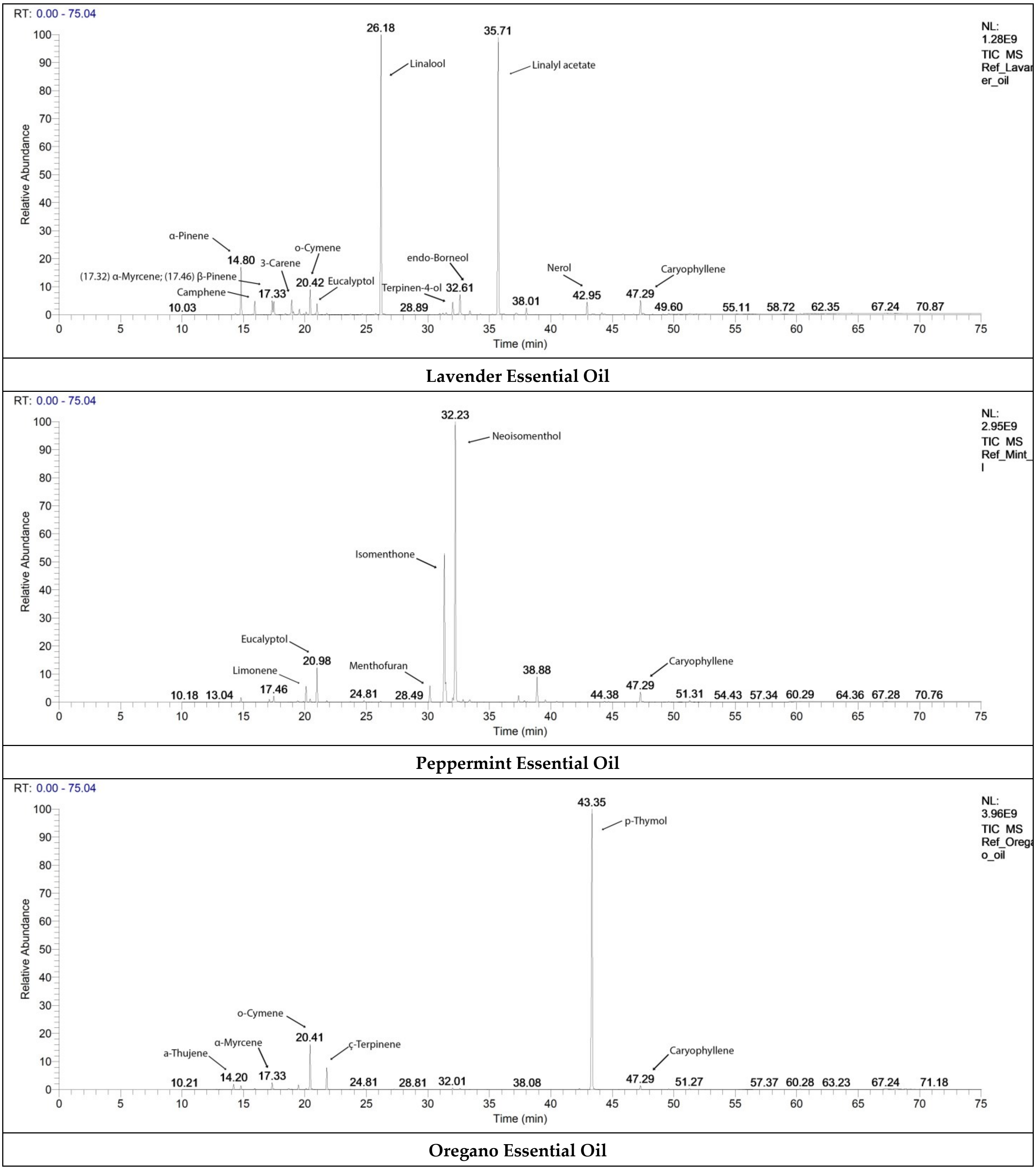

Figure S1. GC-MS Chromatograms of Essential Oils References

Table S1. The Phytochemicals identified and quantified in Essential Oils by GC-MS analysis

RT [min]	Compound name	Clove oil		Eucalyptus oil		Lavender oil		Peppermint oil		Oregano oil	
		Reference [%]	Sample [%]	Reference [%]	Sample [%]	Reference [%]	Sample [%]	Reference [%]	Sample [%]	Reference [%]	Sample [%]
12.83	Furfural	0.0712	0.0507								
14.20	a-Thujene			0.0043	0.0121			0.0280	0.0298	1.1321	0.0121
14.79	α-Pinene			1.7079	2.8165	5.6669	3.1612	0.7020	0.8279	0.8698	2.3971
15.92	Camphene			0.0047	0.0551	1.6620	0.3272	0.0065	0.0058	0.2100	0.0197
16.96	Isocamphane						0.0123				
17.11	Sabinen							0.4209	0.3986		
17.32	α-Myrcene			0.5530	0.3272	1.5522	0.6436	0.0931	0.1196	1.4758	0.0566
17.46	β-Pinene			0.3913	0.2631	1.6132	0.0478	0.9989	0.8802	0.1332	1.3942
18.72	β-Thujene			0.9446	0.4638						
18.92	3-Carene			0.0517	0.0033	1.8368	0.0007			0.0707	
19.47	Terpinolen			0.1711	0.1066						
19.89	α-Ocimene			0.1010	0.0704	0.0672	0.8177	0.0996	0.1088		
20.10	Limonene			6.6187	5.4506	0.2892	1.1219	2.5104	2.0857	0.2360	0.6877
20.42	o-Cymene			4.7347	4.1323	3.0292	0.2470	0.4738	0.1803	10.1224	16.2692
20.57	α-Phellandrene			0.1339	0.1070			0.0208	0.0271		
21.00	Eucalyptol			81.9587	83.7478	1.4208	0.7360	5.9016	5.0452	0.0193	0.8904
21.77	γ-Terpinene			2.6225	2.2315	0.1248	0.0000	0.2195	0.2864	5.1776	2.9827
24.67	Linalool oxide					0.0470	0.0738				
24.81	cis-β-Terpineol									0.1934	
26.16	Linalool				0.0102	35.8533	52.9348	0.1058	0.0670	0.0200	1.4072
28.14	Thujone				0.0113						
28.23	1,2-Dihydrolinalool						0.4868				
30.16	Menthofuran							2.8505	2.4656		
31.21	Lavandulol					0.1657					
31.32	L-Menthone										0.2304
31.34	Isomenthone							29.3657	26.5289		
31.48	Camphor				0.0138	0.2553	1.3463				
32.01	Terpinen-4-ol				0.0373	1.6204	0.6385	0.4541	0.3867	0.4100	0.0163
32.23	Neoisomenthol							50.8339	55.0951		
32.61	endo-Borneol				0.0002	2.7675	0.0107	0.0130	0.0069	0.2792	
32.80	Dihydrocitronellol						0.0246				
33.41	α-Terpineol			0.0018	0.0956	0.4730	0.9501	0.4157	0.3909	0.0633	0.0020
33.76	Terpinyl formate						0.1275				
34.18	Methyl salicylate	0.0411	0.0581								
34.25	(-)-trans-Isopi-peritenol								0.0160		
34.40	Isopulegol acetate						0.0616				

34.54	γ-Terpinen								0.0076		
35.25	Citronellol					0.0247		0.0027	0.0067		
35.44	Dihydrocarvone									0.0161	
35.69	Linalyl acetate					36.2963	32.3146				0.0152
35.85	Menthyl formate							0.0065			
37.16	Geraniol					0.2540					
37.37	Pulegone							1.1453	0.8421		
37.85	Neoisomenthyl ace-tate							0.2541	0.3132		
38.01	Lavandulyl acetate					0.8589					
38.09	Carvone							0.0755	0.0319	0.0108	
39.06	Borneol acetate				0.0430	0.0009					
39.45	Isobornyl acetate						0.1546				
39.55	Anethole				0.0012						
39.55	Isopiperitenone							0.3873	0.9866		
41.23	α-Cubebene					0.0007					
41.77	Chavicol	0.0205	0.0665								
42.31	Carvacrol							0.0218	0.0162	0.2040	
42.95	Nerol					1.6308	2.1132				
43.29	p-Thymol								0.0896	77.9238	72.0862
43.38	Copaene	0.0616	0.0944					0.0100			
44.14	Geranyl acetate					0.2008	0.8031				
44.33	Chavicol acetate		0.0017								
44.38	α-Bourbonene							0.1275	0.2632		
44.70	β-Elemene							0.0490	0.0651		
45.53	Carvacrol acetate									0.0226	
45.79	Eugenol	87.0884	86.2272								
45.87	Santalen					0.0373					
46.62	β-Ylangene							0.0221	0.2274		
47.29	Caryophyllene	9.8462	6.8733			2.0263		1.9040	1.6170	1.0352	1.4581
49.59	Humulene	0.9997	0.6717			0.0930		0.0513	0.0575	0.1180	
50.85	à-Farnesene	0.0121									
51.27	α-Bisabolene									0.2043	
51.30	Germacrene D					0.0894		0.2735	0.4228		
52.58	ç-Muurolene					0.0424				0.0004	
52.60	δ-Cadinene	0.0332	0.0548					0.0275	0.0937		
53.10	trans-α-Bisabolene						0.8443				
53.61	Calamenene		0.0068								
56.43	Eugenol acetate	1.5998	5.7515								
59.14	Caryophyllenyl al-cohol	0.0587									

59.44	Spathulenol							0.0145			
59.88	Mint furanone							0.0482	0.0004		
60.28	Caryophyllene oxide	0.1673	0.1433					0.0657	0.0063	0.0521	0.0748

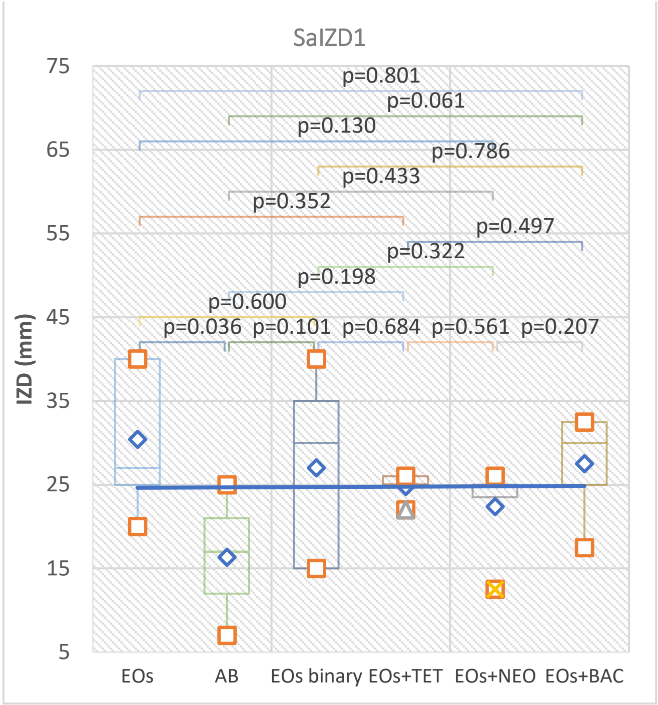

A

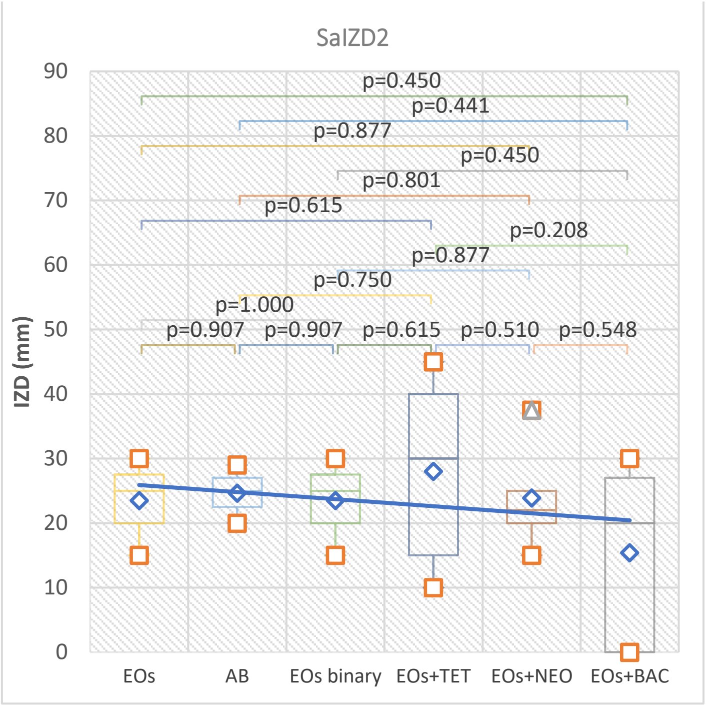

B

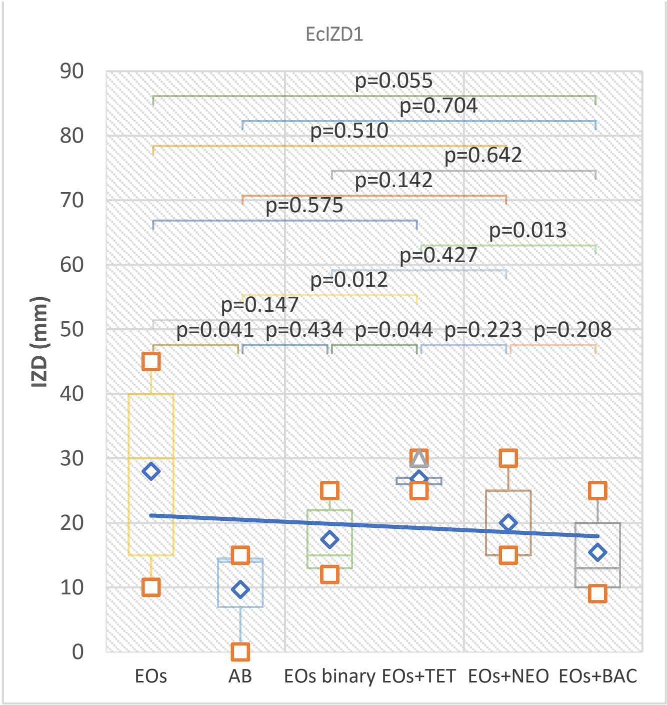

C

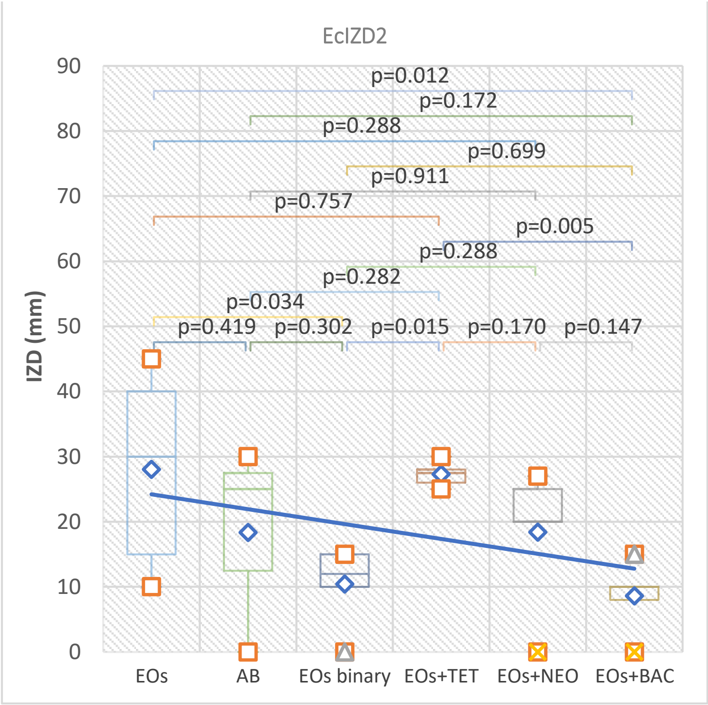

D

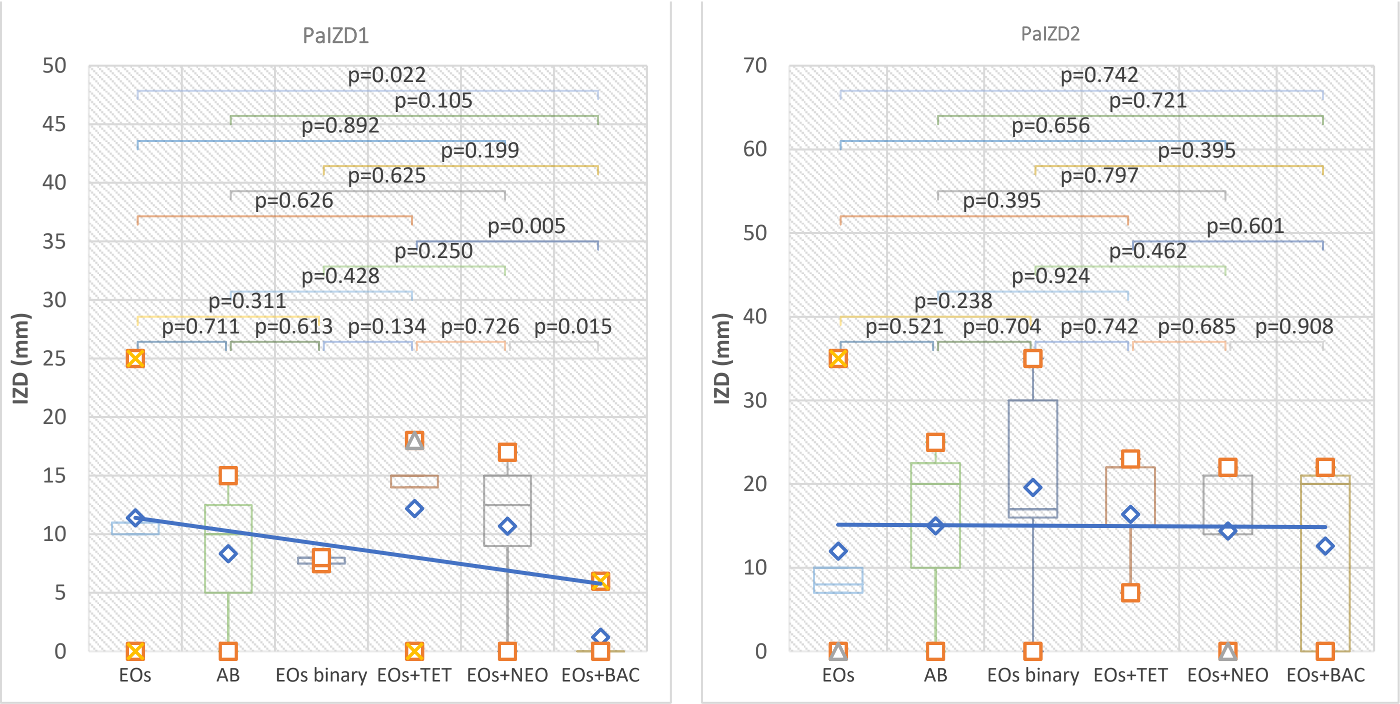

E F

Figure S2. Comparative presentation of antibacterial activities evaluated by both diffusimetric methods (disc diffusion – 1 and cylinder technique -2) for all EOs, conventional antibiotics, Double EOs combinations (EOs binary), Triple combinations (EOs + TET, EOs + NEO, EOs + BAC). **A**. SaIZD1; **B**. SaIZD2; **C.** EcIZD1; **D**. EcIZD2; **E**. PaIZD1; **F**. PaIZD2; NEO—Neomycin; TET—Tetracycline; BAC—Bacitracin; IZD—Inhibition zone diameter (mm), the scale of measurement was as follows: powerful inhibitory effect at IZD ≥ 35 mm, strong inhibitory effects at 35 > IZD ≥ 25 moderate inhibitory effect at 25 > IZD ≥ 15 mm, mild inhibitory effect when 15 > IZD ≥ 10 mm, and no inhibitory effect at IZD < 10 mm. Sa — *S. aureus*, Ec — *E. coli*, Pa — *P. aeruginosa;* Bonferroni corrected significance level is 0.0033 (corresponding to $\alpha = 0.05$).

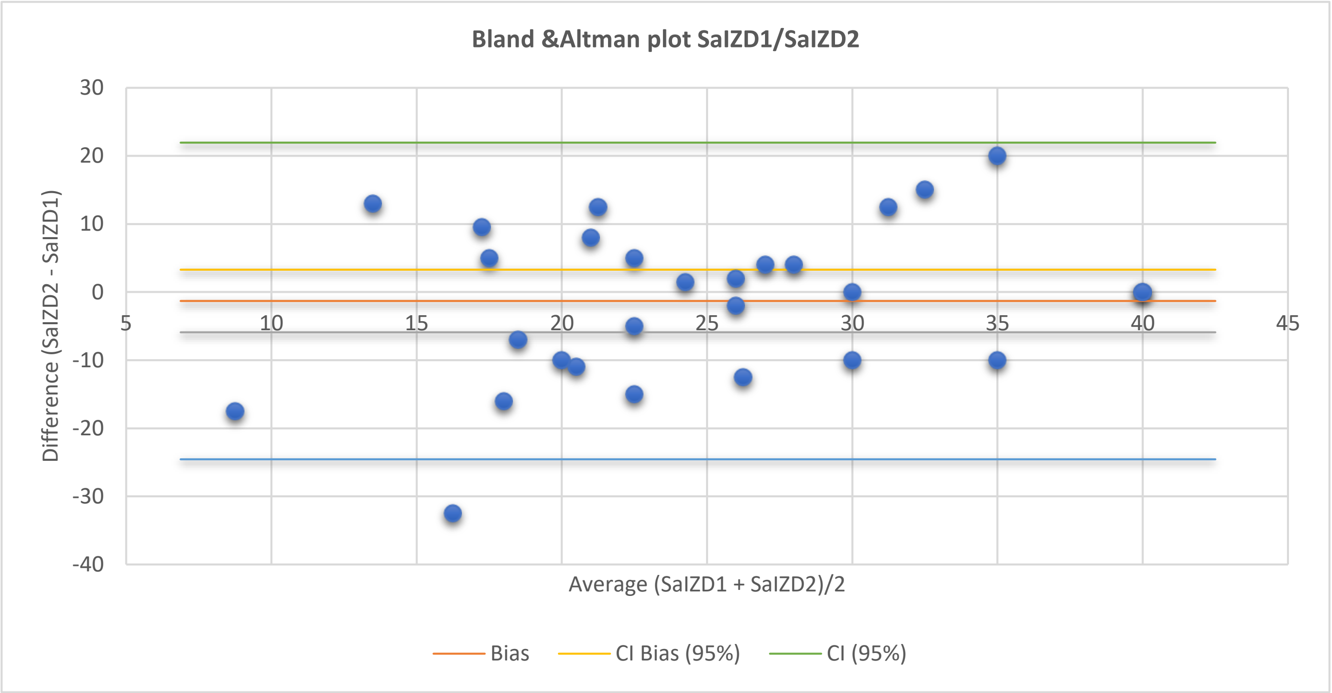
Bland &Altman plot SaIZD1/SaIZD2
30
20
10
0
-10
-20
-30
-40
5
10
15
20
25
30
35
40
45
Difference (SaIZD2 - SaIZD1)
Average (SaIZD1 + SaIZD2)/2
Bias
CI Bias (95%)
CI (95%)

A

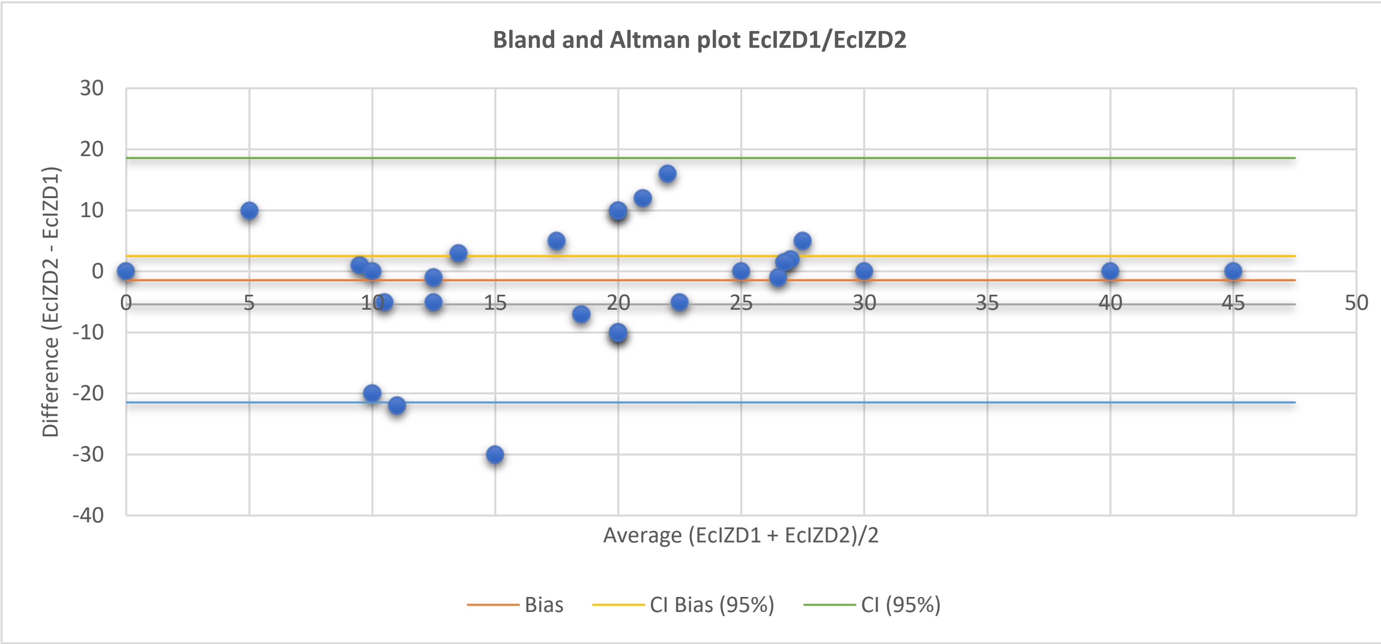
Bland and Altman plot EcIZD1/EcIZD2
30
20
10
0
-10
-20
-30
-40
0
5
10
15
20
25
30
35
40
45
50
Difference (EcIZD2 - EcIZD1)
Average (EcIZD1 + EcIZD2)/2
Bias
CI Bias (95%)
CI (95%)

B

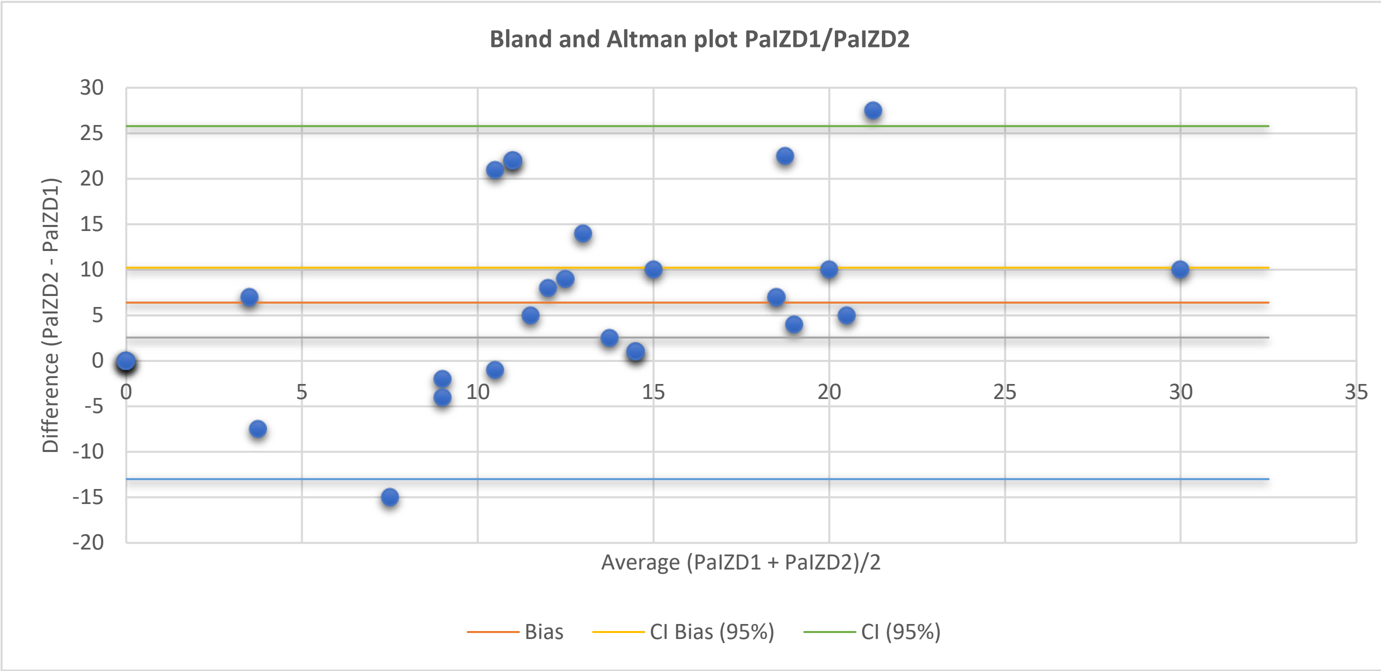

C

S. aureus

t-test for two paired samples / Two-tailed test: SaIZD1/SaIZD2

95% confidence interval on the difference between the means:
] -5.904 ; 3.297 [

Difference	-1.304
t (Observed value)	-0.581
t (Critical value)	2.052
DF	27
p-value (Two-tailed)	0.566
alpha	0.05

E. coli

t-test for two paired samples / Two-tailed test:

95% confidence interval on the difference between the means:
] 2.557; 10.229 [

Difference	6.393
t (Observed value)	3.419
t (Critical value)	2.052
DF	27

p-value (Two-tailed)	0.002
alpha	0.05

p. aeruginosa

t-test for two paired samples / Two-tailed test: PaIZD1/PaIZD2

95% confidence interval on the difference between the means:
] -5.408 ; 2.515 [

Difference	-1.446
t (Observed value)	-0.749
t (Critical value)	2.052
DF	27
p-value (Two-tailed)	0.460
alpha	0.05

Figure S3. Comparison between DDM and CT
